# Preterm birth and maternal heart disease: A machine learning analysis using the Korean national health insurance database

**DOI:** 10.1371/journal.pone.0283959

**Published:** 2023-03-31

**Authors:** Jue Seong Lee, Eun-Saem Choi, Yujin Hwang, Kwang-Sig Lee, Ki Hoon Ahn

**Affiliations:** 1 Department of Pediatric Cardiology, Korea University College of Medicine, Korea University Anam Hospital, Seoul, Korea; 2 Department of Obstetrics and Gynecology, Korea University College of Medicine, Korea University Anam Hospital, Seoul, Korea; 3 AI Center, Korea University College of Medicine, Korea University Anam Hospital, Seoul, Korea; Affiliated Hospital of Nantong University, CHINA

## Abstract

**Background:**

Maternal heart disease is suspected to affect preterm birth (PTB); however, validated studies on the association between maternal heart disease and PTB are still limited. This study aimed to build a prediction model for PTB using machine learning analysis and nationwide population data, and to investigate the association between various maternal heart diseases and PTB.

**Methods:**

A population-based, retrospective cohort study was conducted using data obtained from the Korea National Health Insurance claims database, that included 174,926 primiparous women aged 25–40 years who delivered in 2017. The random forest variable importance was used to identify the major determinants of PTB and test its associations with maternal heart diseases, i.e., arrhythmia, ischemic heart disease (IHD), cardiomyopathy, congestive heart failure, and congenital heart disease first diagnosed before or during pregnancy.

**Results:**

Among the study population, 12,701 women had PTB, and 12,234 women had at least one heart disease. The areas under the receiver-operating-characteristic curves of the random forest with oversampling data were within 88.53 to 95.31. The accuracy range was 89.59 to 95.22. The most critical variables for PTB were socioeconomic status and age. The random forest variable importance indicated the strong associations of PTB with arrhythmia and IHD among the maternal heart diseases. Within the arrhythmia group, atrial fibrillation/flutter was the most significant risk factor for PTB based on the Shapley additive explanation value.

**Conclusions:**

Careful evaluation and management of maternal heart disease during pregnancy would help reduce PTB. Machine learning is an effective prediction model for PTB and the major predictors of PTB included maternal heart disease such as arrhythmia and IHD.

## Introduction

Approximately 15 million neonates are born prematurely (defined as live birth at < 37^0/7^ weeks of gestation) worldwide, accounting for about 11% of global births [1, 2]. The reported rate of preterm birth (PTB) has been increasing in many countries [1, 2]. PTB is the most important cause of death in infants and children, accounting for approximately 18% of deaths in children under the age of five years [1–3]. Cost-effective interventions, particularly focused on controlling maternal risk factors, have been estimated to prevent as much as three quarters of mortality due to PTB [2]. Additionally, identifying maternal PTB risk factors could help us better understand the etiology of PTB.

The number of pregnant women with underlying diseases such as hypertension, diabetes, and obesity increase with maternal aging [4, 5]. This leads to an increased number of pregnant women with heart disease (i.e., ischemic heart disease, cardiomyopathy, or arrhythmia) [4–6]. Furthermore, an increasing number of women with congenital heart disease (CHD) are reaching the reproductive age [4]. Although most women with CHD can carry a pregnancy and deliver safely, there are still concerns [4, 7]. Pregnancy complicated by maternal heart disease is associated with maternal and fetal morbidity and mortality [4, 7]. In addition, both CHD and acquired heart disease are known to affect PTB [4, 7, 8]. In a study of 5,739 pregnant women with acquired heart disease and CHD enrolled in the Registry Of Pregnancy And Cardiac disease (ROPAC) from 2007 to 2018, the prevalence of PTB in mothers with heart disease has been reported to be about 16% [8]. Another German study reported a prevalence of PTB of 11.7% in 2,114 pregnant women with CHD [7]. Overall, it has been consistently reported that the prevalence of PTB is higher in pregnant women with heart disease than in the general population, but there are differences in the prevalence of PTB reported in each country [7–9]. Moreover, most of the reported studies are the results of developed countries in the West, and there are no studies targeting Asian populations yet.

Hence, this study aimed to build a prediction model for PTB using machine learning analysis and nationwide population data, and to investigate the association between various maternal heart diseases and PTB.

## Methods

### Study population

This nationwide population-based cohort study included singleton primiparous women who had delivered in 2017. We restricted the inclusion criteria to primiparous women to adjust prior PTB. Women aged 25–40 years who delivered before 37^0/7^ weeks of gestation were included in the study. Data were extracted from the Korea National Health Insurance Service claims database. The Korean National Health Insurance Service (NHIS) claims data covers almost all citizens of Korea (approximately 50 million) [10]. The Korean NHIS data includes diagnosis codes based on International Classification of Disease, Tenth Revision (ICD-10), demographic information on age, sex, income decile, residential area, etc., and information on medication prescriptions, tests, and procedures performed during outpatient visits or hospitalizations since 2002. For primiparous women who gave birth in 2017, all medical history from 2002, when the Korean NHIS data began to be established, to 2016, the year immediately before delivery, was investigated. A total of 174,926 women were included in the analysis. The study was approved by the Institutional Review Board (IRB) of the Korea University Anam Hospital on November 5, 2018 (no. 2018AN0365). The requirement for informed consent was waived due to the retrospective nature of the study.

### Variables

An explanation of each variable according to the International Classification of Disease, Tenth Revision (ICD-10) code is presented in **S1 Table**. The dependent variable was PTB (birth before 37^0/7^ weeks of gestation) in 2017. Four categories of PTB were introduced according to the ICD-10 code: (1) PTB 1—PTB with preterm premature rupture of membranes (PPROM) only; (2) PTB 2—PTB with spontaneous preterm labor without PPROM; (3) PTB 3—PTB 1 or PTB 2; (4) PTB 4—PTB 3 or other indicated PTB due to maternal or fetal indications. Thirty-six independent variables covered the following information: (1) demographic/socioeconomic determinants in 2017 including age and socioeconomic status measured by an insurance fee with a range of 0 (the lowest group) to 20 (the highest group); (2) obstetric and gynecologic diseases in 2002–2016, namely, gestational diabetes, hypertensive disorders during pregnancy (HDP; including, gestational hypertension, preeclampsia and eclampsia), pelvic inflammatory disease, vaginitis, endometriosis, pelvic organ prolapse, abnormal menstruation, recurrent miscarriage or infertility; (3) heart diseases in 2002–2016, including, CHD (acyanotic CHD, cyanotic CHD, severe lesion, shunt lesion, left or right side lesion, other lesion), arrhythmias (including conduction disorder, Wolff-Parkinson-White [WPW] syndrome, supraventricular tachycardia [SVT], atrial fibrillation/flutter [AF/AFL], ventricular arrhythmia [VA], and sick sinus syndrome [SSS]), cardiomyopathy, congestive heart failure, and ischemic heart disease (IHD); (4) other significant medical histories, including hypertension, diabetes, anemia, hyperlipidemia, pulmonary embolism, endocarditis, sepsis, stroke and cardiac arrest; (5) medication history in 2002–2016, particularly, benzodiazepine, calcium channel blocker (CCB), nitrate, progesterone, hypnotic/sedative drug (antihistamine, zolpidem, eszopiclone, pentobarbital sodium, and benzodiazepine derivates), and tricyclic antidepressant (TCA). These variables were selected based on previous studies and available data [11–13]. These data on disease and medication history were screened using ICD-10 and Anatomical Therapeutic Chemical (ATC) codes, respectively (**S1 and S2 Tables**).

### Statistical analysis

Logistic regression and random forest analyses were used to predict PTB [11–13]. A random forest is a group of decision trees that makes decisions on the dependent variable with a majority vote. A random forest with 100 decision trees was employed in this study: 100 training sets were sampled with replacements, 100 decision trees were trained with the training sets, 100 decision trees made 100 predictions, and the random forest took a majority vote on the dependent variable. The data of all the included observations were split into training and validation sets in an 80:20 ratio (139,940 vs. 34,986 cases). The validation criterion of the trained models was accuracy, which is the ratio of correct predictions among the 34,986 cases. A random forest variable importance was introduced to identify the major determinants of PTB and to test its association with 36 variables. The random forest variable importance of a certain variable (e.g., arrhythmia) can be defined as “the decrease of node impurity (GINI) in case a new branch is created based on the predictor in an average decision tree in the random forest”. Let’s assume that the random forest variable importance of arrhythmia for PTB is 0.0146. This indicates that node impurity (GINI) decreases by 0.0146 in case a new branch is created based on arrhythmia in an average decision tree in the random forest. The performance of the random forest increases as node impurity (GINI) decreases. In this context, the random forest variable importance of arrhythmia measures the contribution of arrhythmia for the performance of the random forest. A variable with the ranking of 18th or higher can be considered to be a major determinant in this study, given that it is a top 50% among 36 variables here. Furthermore, we calculated the Shapley additive explanation (SHAP) values to identify the direction of association between maternal heart disease and PTB in the prediction model. Here, the SHAP value of maternal heart disease measured the difference between the model’s predicted probability of PTB for each participant with and without maternal heart disease. Let’s assume that the SHAP value of atrial fibrillation for PTB is 0.1576. This indicates that the probability of PTB (predicted by the random forest) increases by 0.1576 in case the variable atrial fibrillation is added to the random forest. The SHAP value of atrial fibrillation can be considered to be an equivalence of machine learning to the odds ratio of logistic regression. For the arrhythmia group, which showed an even distribution for the increase or decrease in the risk of PTB in the overall SHAP value analysis, it was assumed that each disease within the category of arrhythmia would have a significantly different effect or mechanism on pregnant women chronically, and a subgroup analysis of arrhythmias was performed. Python (CreateSpace: Scotts Valley, 2009) was employed for the analysis from December 15, 2021 to April 15, 2022.

It needs to be noted that in practice experts in artificial intelligence use random forest variable importance to derive the rankings and values of all predictors for the prediction of the dependent variable. Then, they employ the SHAP plots to evaluate the directions of associations between the predictors and the dependent variable. Linear or logistic regression used to play this role before the SHAP approach took it over. This is because the SHAP approach has a notable strength compared to linear or logistic regression: the former considers all realistic scenarios, un-like the latter. Let us assume that there are three predictors of PTB, i.e., socioeconomic status, age and maternal heart disease. As defined above, the SHAP value of maternal heart disease for PTB for a particular participant is the difference between what machine learning predicts for the prob-ability of PTB with and without maternal heart disease for the participant. Here, the SHAP value for the participant is the average of the following four scenarios for the participant: (1) socioeconomic status excluded, age excluded; (2) socioeconomic status excluded, age included; (3) socio-economic status included, age excluded; and (4) socioeconomic status included, age included. In other words, the SHAP value combines the results of all possible sub-group analyses, which are ignored in linear or logistic regression with an unrealistic assumption of ceteris paribus, i.e., “all the other variables staying constant”.

## Results

### Characteristics of study population

A total of 174,926 women who delivered in 2017 were included in the analysis and 12,701 (7.83%) had preterm birth (PTB 4) (**Table 1**). Among the total study population, 12,234 women had at least one heart disease. Arrhythmia was the most common maternal heart disease, followed by IHD and congestive heart failure (total population incidence: 4.18%, 2.86%, and 0.48% respectively). Hypertension, the major underlying disease for heart disease, was found in 12.36% of study population. The incidence of hypertension, arrhythmia, IHD, cardiomyopathy, and congestive heart failure was significantly higher in women who had PTB than in those who gave birth at term (**Table 1**). The prevalence of PTB in pregnant woman with heart disease is presented in S3 Table. The prevalence of PTB in pregnant women with cardiomyopathy was the highest at 16.0%, and the prevalence of PTB among all pregnant women with heart disease was higher than that among pregnant women without heart disease.

**Table 1:** Baseline characteristics of the study population.

Variables	Term birth (n = 162,225)	Preterm birth (n = 12,701)
Demographic information		
Age at delivery (years)	31.9 (29.4–34.4)	32.0 (29.5–34.5)
Socioeconomic status (Insurance fee)	11.1 (7.6–14.6)	11.0 (7.5–14.5)
Maternal heart diseases		
Arrhythmia	6,713 (4.14)	599 (4.72)
Ischemic heart disease	4,565 (2.81)	442 (3.48)
Cardiomyopathy	79 (0.05)	15 (0.12)
Congestive heart failure	756 (0.47)	90 (0.71)
Cyanotic CHD	36 (0.02)	6 (0.05)
Acyanotic CHD	266 (0.16)	26 (0.21)
Obstetric and gynecologic diseases		
Gestational diabetes	71,482 (44.06)	5,561 (43.78)
Hypertension during pregnancy	6,818 (4.20)	910 (7.16)
Abnormal menstruation	46,680 (28.77)	4,088 (32.19)
Endometriosis	6,776 (4.18)	788 (6.20)
Pelvic inflammatory disease	4,7299 (29.16)	4,325 (34.05)
Recurrent abortion or infertility	35,994 (22.19)	3,971 (31.27)
Vaginitis	129,060 (79.56)	10,393 (81.83)
Other medical diseases		
Hypertension	19726 (12.16)	1825 (14.37)
Diabetes	5961 (3.67)	666 (5.24)
Hyperlipidemia	36805 (22.69)	3364 (26.49)
Anemia	45489 (28.04)	3889 (30.62)
Pulmonary embolism	70 (0.04)	6 (0.05)
Endocarditis	35 (0.02)	1 (0.01)
Sepsis	92646 (57.11)	7623 (60.02)
Stroke	675 (0.42)	77 (0.61)
Cardiac arrest	7 (0.004)	0 (0)
Medication		
Benzodiazepine	68019 (41.93)	5752 (45.29)
Calcium channel blocker	487 (0.30)	60 (0.47)
Nitrate	354 (0.22)	35 (0.28)
Progesterone	26622 (16.41)	2462 (19.38)
Hypnotic/sedative drug	8117 (5.00)	803 (6.32)
Tricyclic antidepressant	16757 (10.33)	1499 (11.80)

Values are median (interquartile range) or n (%).

CHD = congenital heart disease.

### Evaluation metrics of prediction model for PTB

**Table 2** presents the areas under the receiver-operating characteristic curves (AUC) of the random forest. The AUC with oversampling data was 88.53–95.31. Its logistic regression counterparts were within the range 50.10–53.54. The performance measures of the random forest with oversampling data were far beyond those of a logistic regression. Oversampling is an approach that matches the sizes of two groups (participants with and without PTB) to train the machines to balance the two groups. Logistic regression requires an unrealistic assumption of *ceteris paribus*, i.e., “all the other variables staying constant,” which is not required in a random forest. Hence, the findings of the logistic regression are best considered supplementary.

**Table 2:** The areas under the receiver operating characteristic curve (AUC) for the random forest.

**Accuracy**	**PTB 1**	**PTB 2**	**PTB 3**	**PTB 4**
**Original data**				
Logistic regression	0.9442	0.9753	0.9275	0.9243
Random forest	0.9368	0.9725	0.9175	0.9143
**Oversampling**				
Logistic regression	0.6682	0.6760	0.6660	0.6669
Random forest	0.9095	0.9522	0.8961	0.8959
**AUC**	**PTB 1**	**PTB 2**	**PTB 3**	**PTB 4**
**Original data**				
Logistic regression	0.5000	0.5000	0.5000	0.5000
Random forest	0.4997	0.5008	0.4993	0.5000
**Oversampling**				
Logistic regression	0.5010	0.5354	0.5044	0.5089
Random forest	0.9037	0.9531	0.8856	0.8853

PTB 1—PTB with preterm premature rupture of membranes (PPROM) only; PTB 2—PTB with spontaneous preterm labor without PPROM; PTB 3—PTB 1 or PTB 2; PTB 4—PTB 3 or other indicated PTB due to maternal or fetal indications.

AUC = area under the receiver operating characteristic curve; PTB = preterm birth

**Fig 1:**
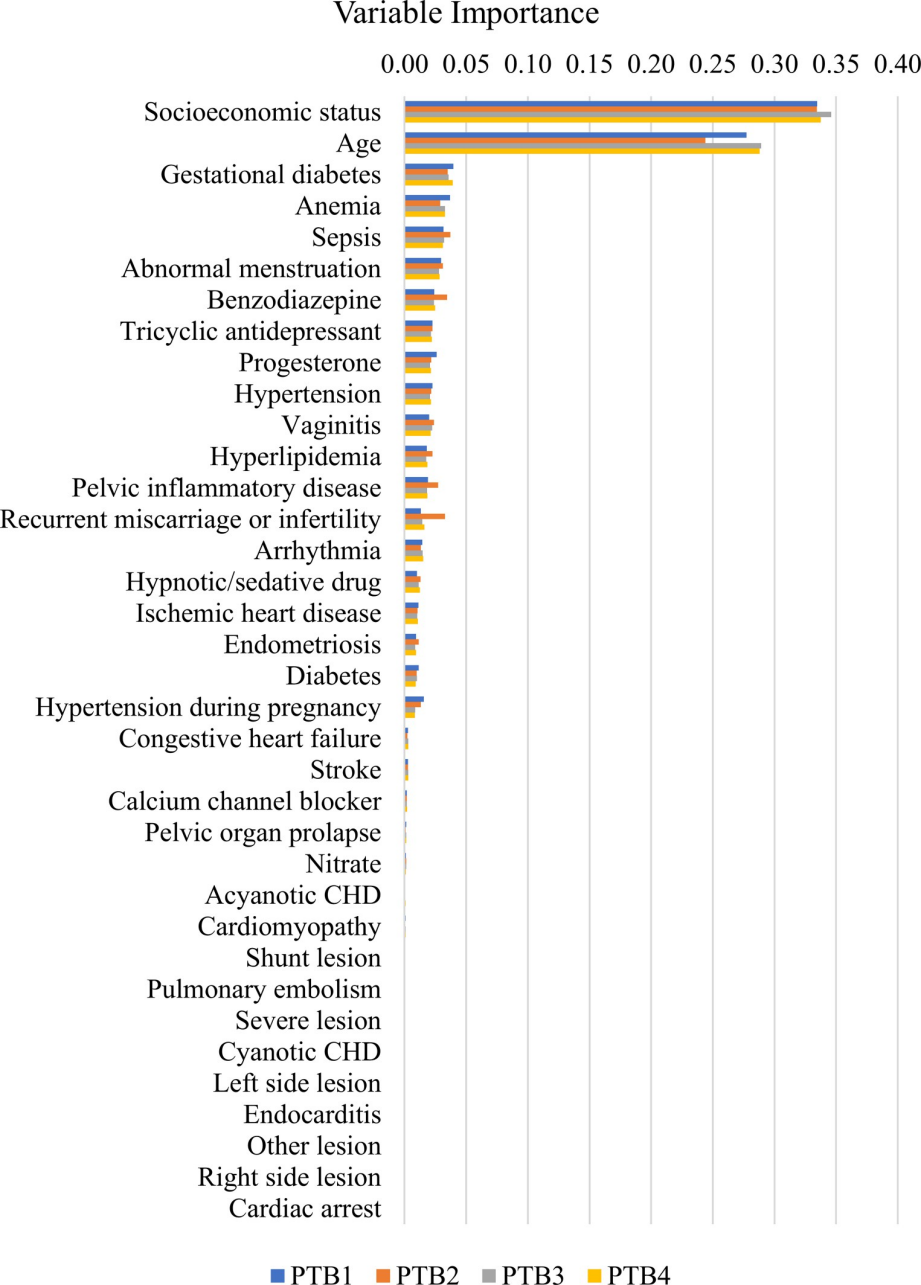
Random forest variable importance for PTB 1–4 (oversampled data). PTB 1—PTB with preterm premature rupture of membranes (PPROM) only; PTB 2—PTB with spontaneous preterm labor without PPROM; PTB 3—PTB 1 or PTB 2; PTB 4—PTB 3 or other indicated PTB due to maternal or fetal indications. PTB = preterm birth; CHD = congenital heart disease.

**Fig 2:**
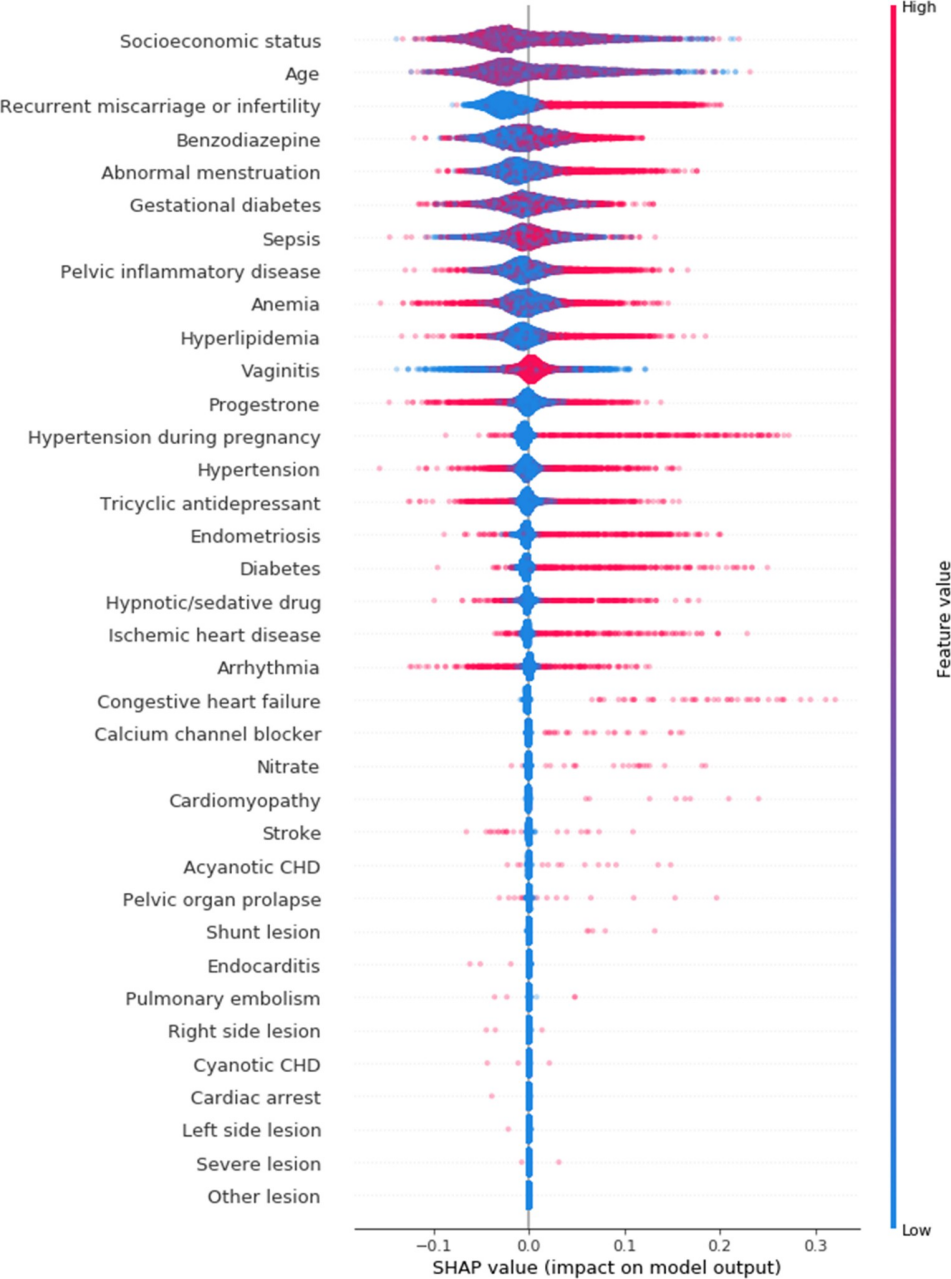
SHAP value of the prediction model for PTB 4. PTB 4 indicated PTB with preterm premature rupture of membranes or spontaneous preterm labor or other indicated PTB due to maternal or fetal indications. PTB = preterm birth; CHD = congenital heart disease.

The random forest variable importance for PTB is shown in **Fig 1**. These values were the averages for PTB 1–4. **Table 3** presents the variable importance of the prediction model for PTB 4. Among the 36 variables, major determinants of PTB were socioeconomic status (0.3377), age (0.2881), gestational diabetes (0.0391), anemia (0.0329), sepsis (0.0311), abnormal menstruation (0.0285), benzodiazepine use (0.0249), TCAs use (0.0221), progesterone use (0.0214), hypertension (0.0213), vaginitis (0.0211), hyperlipidemia (0.0186), pelvic inflammatory disease (0.0184), recurrent miscarriage or infertility (0.0162), arrhythmia (0.0146), hypnotic/sedative drugs (0.0124), and IHD (0.0107). The variable importance of the prediction model for PTB 1–3 is presented in **S3 Table**. It should be noted that the variable importance measures of the random forest for the oversampling data were very similar to those for the original data (**Table 3 and S4 Table**). Notably, the SHAP value in **Fig 2** shows the sign and magnitude of the effect of major determinants on PTB. For instance, the presence of recurrent miscarriages/infertility was consistently associated with an increased risk of PTB. In contrast, though anemia had a significant effect on PTB (**Table 3**), the direction of the effect was inconsistent (**Fig 2**).

**Table 3:** Random forest variable importance of prediction model for PTB 4. PTB 4 indicated PTB with preterm premature rupture of membranes or spontaneous preterm labor or other indicated PTB due to maternal or fetal indications.

(A) Variable importance in original data		
**Rank**	**PTB4**	**Variable importance**
1	Socioeconomic status	0.3414
2	Age	0.2781
3	Gestational diabetes	0.0382
4	Anemia	0.0352
5	Sepsis	0.0324
6	Abnormal menstruation	0.0298
7	Benzodiazepine	0.0269
8	Hyperlipidemia	0.0225
9	Progesterone	0.0222
10	Hypertension	0.0218
11	Tricyclic antidepressant	0.0212
12	Pelvic inflammatory disease	0.0209
13	Vaginitis	0.0204
14	Arrhythmia	0.0146
15	Hypnotic/sedative drug	0.0119
16	Recurrent miscarriage or infertility	0.0111
17	Diabetes	0.0105
18	Ischemic heart disease	0.0103
19	Endometriosis	0.0093
20	Hypertension during pregnancy	0.0072
21	Stroke	0.0032
22	Congestive heart failure	0.0031
23	Calcium channel blocker	0.0020
24	Nitrate	0.0012
25	Pelvic organ prolapse	0.0012
26	Cardiomyopathy	0.0010
27	Acyanotic CHD	0.0007
28	Pulmonary embolism	0.0005
29	Shunt lesion	0.0004
30	Severe lesion	0.0002
31	Cyanotic CHD	0.0002
32	Left side lesion	0.0001
33	Endocarditis	0.0001
34	Right side lesion	0.0001
35	Other lesions	0.0000
36	Cardiac arrest	0.0000
(B) Variable importance in oversampled data		
**Rank**	**PTB4**	**Variable importance**
1	Socioeconomic status	0.3377
2	Age	0.2881
3	Gestational diabetes	0.0391
4	Anemia	0.0329
5	Sepsis	0.0311
6	Abnormal menstruation	0.0285
7	Benzodiazepine	0.0249
8	Tricyclic antidepressant	0.0221
9	Progesterone	0.0214
10	Hypertension	0.0213
11	Vaginitis	0.0211
12	Hyperlipidemia	0.0186
13	Pelvic inflammatory disease	0.0184
14	Recurrent miscarriage or infertility	0.0162
15	Arrhythmia	0.0152
16	Hypnotic/sedative drug	0.0124
17	Ischemic heart disease	0.0107
18	Endometriosis	0.0094
19	Diabetes	0.0090
20	Hypertension during pregnancy	0.0084
21	Congestive heart failure	0.0031
22	Stroke	0.0031
23	Calcium channel blocker	0.0020
24	Pelvic organ prolapse	0.0013
25	Nitrate	0.0012
26	Acyanotic CHD	0.0008
27	Cardiomyopathy	0.0008
28	Shunt lesion	0.0004
29	Pulmonary embolism	0.0003
30	Severe lesion	0.0002
31	Cyanotic CHD	0.0002
32	Left side lesion	0.0001
33	Endocarditis	0.0001
34	Other lesions	0.0001
35	Right side lesion	0.0000
36	Cardiac arrest	0.0000

PTB = preterm birth; CHD = congenital heart disease.

### Association between maternal heart disease and PTB

**Fig 3:**
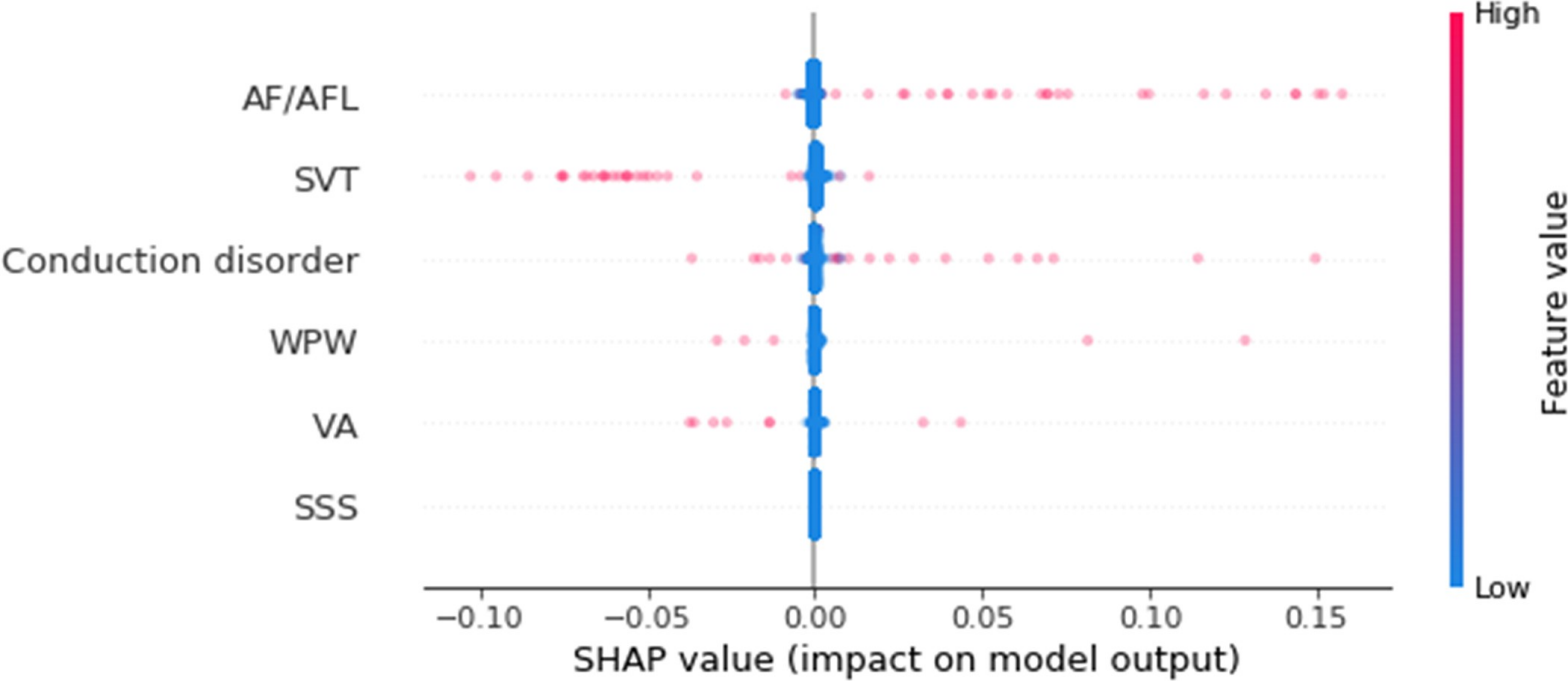
SHAP value of arrhythmia subgroup. AF = atrial fibrillation; AFL = atrial flutter; SVT = supraventricular tachycardia; WPW = Wolff-Parkinson-White syndrome; VA = ventricular arrhythmia; SSS = sick sinus syndrome.

Among the maternal heart diseases, arrhythmia (ranked 15^th^ on variable importance) was the most significant determinant of PTB, followed by IHD (17^th^), congestive heart failure (21^st^), acyanotic CHD (26^th^), and cardiomyopathy (27^th^), in that order. Based on SHAP values, the presence of IHD, congestive heart failure, and cardiomyopathy was associated with an increased PTB risk (**Fig 2 and S5 Table**). Although the variable importance of IHD was lower than that of hypertension, the presence of IHD more consistently increased the risk of PTB than hypertension. On the other hand, the presence of arrhythmia affected both the increasing and decreasing risk of PTB according to the SHAP value. To further delineate the effect of arrhythmia on PTB, we analyzed the arrhythmia subgroups. The subgroups included in the analysis were SVT, AF/AFL, conduction disorder, WPW syndrome, VA, and SSS. The incidence of maternal conduction disorders and AF/AFL was higher in the PTB group than in the term birth group (**Table 4**). Based on the SHAP values, AF/AFL and conduction disorders particularly increased the risk of PTB among arrhythmia subgroups (**Fig 3 and S6 Table**).

**Table 4:** Subgroup analysis of arrhythmia.

Variables	Term birth (n = 162,225)	Preterm birth (n = 12,701)	*P* value
AF/AFL	584 (0.36)	68 (0.54)	0.0018
SVT	599 (0.37)	55 (0.43)	0.2566
Conduction disorder	378 (0.23)	44 (0.35)	0.0121
WPW syndrome	62 (0.04)	6 (0.05)	0.6194
VA	116 (0.07)	7 (0.06)	0.5021
SSS	28 (0.02)	3 (0.02)	0.6041

Values are n (%).

AF = atrial fibrillation; AFL = atrial flutter; SVT = supraventricular tachycardia; WPW = Wolff-Parkinson-White syndrome; VA = ventricular arrhythmia; SSS = sick sinus syndrome.

## Discussion

This study presents a comprehensive analysis of the determinants of PTB, using a population-based cohort of 174,926 participants and a rich collection of 36 variables, including sociodemographic factors, maternal heart disease, obstetric and gynecologic diseases, and other medical history. Using machine learning analysis, we established a validated prediction model for PTB, and investigated the association between various maternal heart diseases and PTB. The AUC of the random forest with oversampling data was within the range 88.53–95.31 and the accuracy was 89.59–95.22. Variable importance for PTB 1–4 showed similar results, and the analysis was focused on PTB 4, the most comprehensive concept among them. The most critical variables for PTB were socioeconomic status and age. The major determinants of PTB among the maternal heart diseases were arrhythmia and IHD. In the SHAP value analysis, congestive heart failure, cardiomyopathy, and IHD were associated with an increased risk of PTB. Within the arrhythmia subgroups, AF/AFL and conduction disorders were associated with an increased risk of PTB.

Different studies used different variables and machine learning models to predict PTB [14–19]. For the prediction of PTB, for example, a retrospective study used five machine learning models and a population-based birth cohort in Western Australia during 1980–2015. This study covered a great variety of maternal demographic, socioeconomic, obstetric and medical variables to register the AUC of 0.56–0.86 for the prediction of PTB [14]. On the contrary, a prospective study focused on two variables, i.e., cervical length and quantitative fetal fibronectin, for the prediction of PTB in 1803 asymptotic women in 13 UK birth clinics. This study employed machine learning-based survival analysis and reported the AUC of 0.96 for PTB of less than 30 weeks and 0.77 for PTB less than 37 weeks [18]. A recent study would be positioned between these two extremes: This study included nine intrauterine and extrauterine variables for PTB, i.e., placenta previa, pregnancy-induced hypertension, antibiotics, cervix length, physical exercise, fetal growth, maternal anxiety, preeclampsia and antihypertensives. This study utilized these variables and the random forest to achieve the accuracy of 81.08% and the AUC of 81.22% [19]. We used the random forest and considered a large collection of 36 demographic, socioeconomic, obstetric and medical variables to record the highest AUC of 0.95 for the prediction of PTB. Socioeconomic status and age were found to be the most important variables in this study. We also paid special attention to the association between maternal heart disease and PTB, given that maternal heart disease was discovered to be an important variable for PTB in this study. This finding is consistent with that of a previous machine learning study stating that maternal cardiovascular disease is an important variable for PTB [16]. Indeed, we furthered this line of research by exploring the relationship of PTB with each of maternal heart diseases.

### Effect of maternal heart disease on PTB

There are possible hypotheses for the association between maternal heart disease and PTB. First, in pregnant women with heart disease, cardiac adaptation following conception differs from that in healthy women [4, 20]. Usually, cardiac output increases by 30–50% above the baseline by 32 weeks of gestation [4, 20]. However, in pregnant women with underlying heart diseases, the increase in cardiac output becomes suboptimal, which could affect the uteroplacental blood flow [20, 21]. Second, pregnant women with heart disease are more likely to have a variety of cardiovascular risk factors (hypertension, diabetes, obesity, hyperlipidemia, etc.) [4, 6]. These risk factors also contribute to the increased risk of PTB [22–24]. Third, cardiovascular medications may have affected PTB. Some cardiovascular drugs are known to affect PTB, but the data are still limited [4, 6, 25].

### Arrhythmia and PTB

In this study, arrhythmia was a major determinant of PTB. In particular, AF/AFL and conduction disorders showed a positive correlation with PTB. Women with prenatal arrhythmias are more likely to develop arrhythmia episodes during pregnancy [26, 27]. It has been reported that approximately 50% of mothers with prenatal AF/AFL may experience recurrent episodes during pregnancy [26, 27]. Even in mothers without prenatal arrhythmias, new-onset arrhythmias can occur during pregnancy due to hemodynamic, hormonal, and autonomic changes [26]. Previous studies have reported that arrhythmias during pregnancy can increase PTB due to uteroplacental insufficiency and fetal hypoxia [27, 28]. In addition, antiarrhythmic drugs or anticoagulants may have had an effect on the development of PTB, but the evidence is still limited [25, 27].

### IHD and PTB

IHD was ranked 17^th^ in variable importance and second among maternal heart diseases. IHD was positively correlated with PTB in the SHAP value. Endothelial dysfunction, a known key player in the pathophysiology of IHD [29], induces inflammation and thrombosis which are the precursors of both IHD and PTB [24, 29, 30]. In addition, a study has reported that the biomarkers of endothelial dysfunction, such as soluble intercellular adhesion molecule-1 were elevated in the women with PTB [31]. And mothers with IHD are more likely to have underlying diseases such as diabetes, hypertension, and these underlying diseases may also have affected the increase in PTB [22, 23].

### Heart failure/Cardiomyopathy and PTB

Congestive heart failure is ranked 21^st^ in variable importance and third among heart diseases. The SHAP value showed the most significant positive correlation between cardiomyopathy and PTB among all the variables. Cardiomyopathy was ranked 27^th^ in variable importance and fourth among the heart diseases. The SHAP values showed a consistently positive correlation between cardiomyopathy and PTB. Heart failure is the most common complication experienced during pregnancy by mothers with pre-existing heart disease [32]. In particular, patients with cardiomyopathy commonly experience the occurrence and exacerbation of heart failure during pregnancy [32]. Several studies have reported that maternal heart failure is associated with an increased risk of PTB [32, 33]. Comparable to previous studies, this study used a national database and machine learning and thus, showed a consistent association between heart failure and PTB.

### CHD and PTB

The variable importance of CHD was relatively lower than that of the other maternal heart diseases, such as arrhythmia or IHD. Although women with CHD are known to have favorable pregnancy outcomes, the risk of adverse outcomes, including PTB, has been reported to increase depending on the CHD severity or lesion characteristics [4, 7, 21]. In this cohort, only 334 women with CHD (0.2%) were pregnant in Korea in 2017, and 42 of them had cyanotic CHD (0.02%). PTB 4 occurred in 26 patients with acyanotic CHD (8.9%) and six patients with cyanotic CHD (14.3%). The relatively low number of patients with CHD probably caused the unexpectedly low variable importance of CHD. Additionally, it is presumed that those in a relatively healthy condition became pregnant, contributing to the low variable importance of CHD. Nevertheless, the incidence of PTB in mothers with acyanotic (8.9%) and cyanotic CHD (14.3%) was higher than in mothers with arrhythmia (8.2%) or IHD (8.8%); therefore, caution about PTB in patients with CHD should not be overlooked. Moreover, fetal CHD as well as maternal CHD may be one of the major factors affecting PTB. Giorgione et al. [34] reported an adjusted odds ratio of 2.17 (95% CI, 1.24–3.81) for PTB in fetal CHD cases. As a possible explanation for this, maternal placental dysfunction or imbalances in placental angiogenic factors have been suggested as parameters that simultaneously affect PTB and fetal CHD [34, 35]. Actually, fetal CHD could not be identified in our data and therefore could not be included in the analysis, and more research on this issue will be needed in the future.

### Hypertension and PTB

In this study, hypertension, which is the main underlying disease of heart disease, was a major determinant of PTB (ranked 10^th^). This is comparable to the results of previous studies [22, 23, 36]. Pre-gestational hypertension is noted as a risk factor for PTB [22, 23, 36]. Besides the common risk factors that hypertension and PTB share, the association between hypertension and (superimposed) preeclampsia also contributes to this finding [22–24, 36]. Pre-gestational hypertension increases the risk of (superimposed) preeclampsia [36]. Moreover, women with hypertension tend to have a more severe form and earlier onset of preeclampsia than those without [37].

In our study, age and socioeconomic status were found to be the most important variables for predicting PTB, while the importance of maternal heart disease as a predictor was relatively low compared to these factors. This may be due to the relatively low frequency of maternal heart disease in the overall sample. However, as previously mentioned, it has consistently been reported that the risk of PTB is significantly increased in pregnant women with heart disease. Therefore, reducing the risk of PTB in mothers with heart disease is an important issue, and our study results may have important implications in this regard. Variables such as socioeconomic status, age, and gestational diabetes, which showed the highest variable importance in our machine learning analysis, may also increase the risk of PTB in pregnant women with heart disease. The impact of these variables on the risk of PTB in high-risk groups such as pregnant women with heart disease may be even more significant. Therefore, if we explore ways to control these factors in pregnant women with heart disease, it may contribute to reducing the risk of PTB in pregnant women with heart disease in the future.

The prevalence of maternal heart disease and its risk factors have increased over the past decades [4–6]. This study verified that, among maternal heart diseases, arrhythmia and IHD are major determinants of PTB. Among the arrhythmias, there was a significant correlation between PTB and AF/AFL and conduction disorders. There was an association between PTB and heart failure/cardiomyopathy and CHD, in that order. To our knowledge, this is the first study that used a large, population database and machine learning to evaluate the importance of various heart diseases in PTB. Evaluation and management of maternal heart disease may help reduce PTB and improve neonatal outcomes. Further research is needed to identify the ideal management or intervention to improve pregnancy outcomes in women with heart disease.

### Limitations

This study had some limitations. First, this study did not examine the possible mediating effects among the variables (e.g., the mediating effects of socioeconomic status between heart disease and preterm birth). Second, a recent review suggested that different machine learning approaches would be optimal for different types of data regarding the prediction of PTB: the artificial neural network, logistic regression, and/or random forest for numeric data; the support vector machine for electrohysterogram data; the recurrent neural network for textual data; and the convolutional neural network for imaging data [12]. Integrating various kinds of machine learning approaches for various kinds of PTB data would bring new innovations and deeper insights into this line of research. Third, we did not perform the subgroup analysis of PTB in this study. PTB is divided according to the cause of PTB or gestational age. The classification according to the cause of PTB, there are indicated PTB (PTB caused by preterm labor or PPROM) and spontaneous PTB (PTB induced because of the maternal-fetal condition such as severe preeclampsia or non-reassuring fetal heart rate) and according to gestational age, there are early PTB (born before 32^0/7^ weeks of gestation) and late PTB (born at 32^1/7^ weeks to 36^6/7^ weeks of gestation) [38, 39]. The Korean NHIS claims data does not provide the clinical information regarding the cause of PTB. Therefore, the current study could not differentiate the spontaneous PTB and indicated PTB. Because the pathophysiology of each PTB is different, the risk factors would be different. Further research focusing on the spontaneous PTB could improve identifying the association between maternal heart disease and PTB. Likewise, we did not subdivide PTB according to the gestational age (early PTB, born before 34^0/7^ weeks of gestation; late preterm birth, born at 34^1/7^ weeks to 36^6/7^ weeks of gestation) in this study. Severe morbidities and higher mortality in early preterm neonates than in the late preterm neonates, makes analyzing the rates of early and late PTB important as it could provide more detailed information. We plan to do follow-up studies considering these limitations of this study. Fourth, when initially constructing the dataset, the data was constructed from 25 to 40 years of age, considering the total data size. However, this age restriction may exclude important information and introduce errors into the analysis. Finally, we included a total of 36 variables by trying to include as many variables as possible among the variables that can be identified in the Korea National Health Insurance Service claims database. This includes obstetrics and gynecological diseases and drugs that have been identified as affecting PTB in our previous study [11–13]. However, there were variables that could not be identified with this data, such as prior PTB, short cervical length, fetal growth restriction, obesity, smoking, and alcohol consumption, and there may be other confounding variables that may have influenced PTB, but we have not identified. Nevertheless, we attempted to comprehensively analyze the association between various heart diseases and PTB through machine learning analysis. Even with some limitations due to some of the lacking variables, The AUC of the prediction model was within the range 88.53–95.31 and the accuracy was 89.59–95.22, showing a high validity.

## Conclusion

Machine learning is an effective prediction model for PTB and the major predictors of PTB included maternal heart disease such as arrhythmia and IHD. We used the random forest and considered a large collection of 36 demographic, socioeconomic, obstetric and medical variables to record the highest AUC of 0.95 for the prediction of PTB. Careful evaluation and management of maternal heart disease during pregnancy would help reduce PTB. Further research is needed on this strategy.

## Supporting information

S1 Table ICD-10 code for each variable. (DOCX)

S2 Table ATC code for medication. (DOCX)

S3 Table The prevalence of PTB in pregnant woman with and without heart disease. (DOCX)

S4 Table Variable importance in random forest prediction model for PTB 1–3. (DOCX)

S5 Table SHAP range for PTB 4. (DOCX)

S6 Table SHAP range of the arrhythmia subgroup. (DOCX)

## Abbreviations list

PTB: preterm birth
CHD: congenital heart disease
PPROM: preterm premature rupture of membranes
HDP: hypertensive disorders during pregnancy
WPW: Wolff-Parkinson-White
SVT: supraventricular tachycardia
AF: atrial fibrillation
AFL: atrial flutter
VA: ventricular arrhythmia
SSS: sick sinus syndrome
IHD: ischemic heart disease
CCB: calcium channel blocker
TCA: tricyclic antidepressant
SHAP: Shapley additive explanations
