# Supplementary material for: Preterm birth and maternal heart disease: A machine learning analysis using the Korean national health insurance database
Source: PLoS One. 2023 Mar 31;18(3):e0283959. doi: 10.1371/journal.pone.0283959 (PMC10065252; doi:10.1371/journal.pone.0283959)
Supplement: S1 Table — (DOCX) [file pone.0283959.s001.docx]

**S1 Table. ICD-10 code for each variable**

|  |  | **Code** | **Description** |
| --- | --- | --- | --- |
| PTB | PTB1  (PTB with PPROM) | O42.00 | Onset of labor within 24 hours of rupture (0-33 weeks of gestation) |
|  |  | O42.01 | Onset of labor within 24 hours of rupture (34-36 weeks of gestation) |
|  |  | O42.10 | Onset of labor after 24 hours of rupture (0-33 weeks of gestation) |
|  |  | O42.11 | Onset of labor after 24 hours of rupture (34-36 weeks of gestation) |
|  |  | O42.20 | Prolonged labor because of treatment (0-33 weeks of gestation) |
|  |  | O42.21 | Prolonged labor because of treatment (34-36 weeks of gestation) |
|  |  | O42.90 | Unspecified length of time between rupture and onset of labor (0-33 weeks of gestation) |
|  |  | O42.91 | Unspecified length of time between rupture and onset of labor (34-36 weeks of gestation) |
|  | PTB2  (PTB with spontaneous labor without PPROM) | O60.11 | Preterm delivery with preterm labor (0-33 weeks of gestation) |
|  |  | O60.12 | Preterm delivery with preterm labor (34-36 weeks of gestation) |
|  |  | O60.19 | Preterm delivery with preterm labor (unspecified weeks of gestation) |
|  | Other indicated PTB | O60.31 | Preterm delivery without preterm labor (0-33 weeks of gestation) |
|  |  | O60.32 | Preterm delivery without preterm labor (34-36 weeks of gestation) |
|  |  | O60.39 | Preterm delivery without preterm labor (unspecified weeks of gestation) |
| Pelvic inflammatory disease | | N73  N74 | Other female pelvic inflammatory diseases  Female pelvic inflammatory disorders in diseases classified elsewhere |
| Vaginitis | | N76 | Other inflammation of vagina and vulva |
| Endometriosis | | N80 | Endometriosis |
| Pelvic organ prolapse | | N81 | Female genital prolapse |
| Abnormal menstruation | | N91.4 | Secondary oligomenorrhoea |
|  |  | N91.5 | Oligomenorrhoea, unspecified |
|  |  | N92.0 | Excessive and frequent menstruation with regular cycle |
|  |  | N92.1 | Excessive and frequent menstruation with irregular cycle |
|  |  | N92.3 | Ovulation bleeding |
|  |  | N92.4 | Excessive bleeding in the premenopausal period |
|  |  | N92.5 | Other specified irregular menstruation |
|  |  | N92.6 | Irregular menstruation, unspecified |
| Recurrent miscarriage or infertility | | N96 | Habitual abortion |
|  |  | N97 | Female infertility |
|  |  | N98 | Complications associated with artificial fertilization |
| Anemia | | D50 | Iron deficiency anemia |
|  |  | D51 | Vitamin B12 deficiency anemia |
|  |  | D52 | Folate deficiency anemia |
|  |  | D53 | Other nutritional anemias |
|  |  | D55 | Anemia due to enzyme disorders |
|  |  | D57 | Sickle-cell disorders |
|  |  | D58 | Other hereditary hemolytic anemias |
|  |  | D59 | Acquired hemolytic anemia |
|  |  | D60 | Acquired pure red cell aplasia |
|  |  | D61 | Other aplastic anemias and other bone marrow failure syndromes |
|  |  | D62 | Acute post-hemorrhagic anemia |
|  |  | D63 | Anemia in chronic diseases classified elsewhere |
|  |  | D64 | Other anemias |
| Acyanotic congenital heart disease | | Q21.0 | Ventricular septal defect |
|  |  | Q21.1 | Atrial septal defect |
|  |  | Q21.2 | Atrioventricular septal defect |
|  |  | Q21.4 | Aortopulmonary window |
|  |  | Q21.9 | Congenital malformation of cardiac septum, unspecified |
|  |  | Q22.1 | Congenital pulmonary valve stenosis |
|  |  | Q22.2 | Congenital pulmonary valve insufficiency |
|  |  | Q22.3 | Other congenital malformations of pulmonary valve |
|  |  | Q24.3 | Pulmonary infundibular stenosis |
|  |  | Q25.6 | Stenosis of pulmonary artery |
|  |  | Q25.7 | Other congenital malformations of pulmonary artery |
|  |  | Q22.8 | Other congenital malformations of tricuspid valve |
|  |  | Q22.9 | Congenital malformation of tricuspid valve, unspecified |
|  |  | Q23.0 | Congenital stenosis of aortic valve |
|  |  | Q23.1 | Congenital insufficiency of aortic valve |
|  |  | Q24.4 | Congenital subaortic stenosis |
|  |  | Q25.1 | Coarctation of aorta |
|  |  | Q25.2 | Atresia of aorta |
|  |  | Q25.3 | Stenosis of aorta |
|  |  | Q25.4 | Other congenital malformations of aorta |
|  |  | Q23.2 | Congenital mitral stenosis |
|  |  | Q23.3 | Congenital mitral insufficiency |
|  |  | Q23.8 | Other congenital malformations of aortic and mitral valves |
|  |  | Q23.9 | Congenital malformation of aortic and mitral valves, unspecified |
|  |  | Q24.2 | Cor triatriatum |
|  |  | Q24.5 | Malformation of coronary vessels |
|  |  | Q25.0 | Patent ductus arteriosus |
|  |  | Q25.6 | Stenosis of pulmonary artery |
|  |  | Q25.7 | Other congenital malformations of pulmonary artery |
|  |  | Q25.8 | Other congenital malformations of great arteries |
|  |  | Q25.9 | Congenital malformation of great arteries, unspecified |
|  |  | Q26.3 | Partial anomalous pulmonary venous connection |
|  |  | Q26.4 | Anomalous pulmonary venous connection, unspecified |
| Cyanotic congenital heart disease | | Q20.0 | Common arterial trunk |
|  |  | Q20.1 | Double outlet right ventricle |
|  |  | Q20.2 | Double outlet left ventricle |
|  |  | Q20.3 | Discordant ventriculoarterial connection |
|  |  | Q20.4 | Double inlet ventricle |
|  |  | Q20.5 | Discordant atrioventricular connection |
|  |  | Q21.3 | Tetralogy of Fallot |
|  |  | Q21.8 | Other congenital malformations of cardiac septa |
|  |  | I27.8 | Other specified pulmonary heart diseases |
|  |  | Q22.0 | Pulmonary valve atresia |
|  |  | Q22.4 | Congenital tricuspid stenosis, atresia |
|  |  | Q22.6 | Hypoplastic right heart syndrome |
|  |  | Q25.5 | Atresia of pulmonary artery |
|  |  | Q22.5 | Ebstein anomaly |
|  |  | Q23.4 | Hypoplastic left heart syndrome |
|  |  | Q26.0 | Congenital stenosis of vena cava |
|  |  | Q26.8 | Other congenital malformations of great veins |
|  |  | Q26.9 | Congenital malformation of great vein, unspecified |
|  |  | Q26.2 | Total anomalous pulmonary venous connection |
| Severe lesion (of congenital heart disease) | | Q21.2 | Atrioventricular septal defect |
|  |  | Q20.4 | Double inlet ventricle |
|  |  | Q21.3 | Tetralogy of Fallot |
|  |  | Q20.3 | Discordant ventriculoarterial connection |
|  |  | Q20.5 | Discordant atrioventricular connection |
|  |  | Q20.0 | Common arterial trunk |
|  |  | Q21.8 | Other congenital malformations of cardiac septa |
|  |  | I27.8 | Other specified pulmonary heart diseases (Eisenmenger’s syndrome) |
| Shunt lesion (of congenital heart disease) | | Q21.1 | Atrial septal defect |
|  |  | Q21.0 | Ventricular septal defect |
|  |  | Q25.0 | Patent ductus arteriosus |
|  |  | Q21.2 | Atrioventricular septal defect |
|  |  | Q21.4 | Aortopulmonary septal defect |
| Left side lesion (of congenital heart disease) | | Q23.2 | Congenital mitral stenosis |
|  |  | Q23.3 | Congenital mitral insufficiency |
|  |  | Q23.0 | Congenital stenosis of aortic valve |
|  |  | Q24.4 | Congenital subaortic stenosis |
|  |  | Q25.3 | Stenosis of aorta |
|  |  | Q25.4 | Other congenital malformations of aorta |
|  |  | Q23.1 | Congenital insufficiency of aortic valve |
|  |  | Q25.1 | Coarctation of aorta |
|  |  | Q25.2 | Atresia of aorta |
|  |  | Q23.8 | Other congenital malformations of aortic and mitral valves |
|  |  | Q23.9 | Congenital malformation of aortic and mitral valves, unspecified |
|  |  | Q23.4 | Hypoplastic left heart syndrome |
| Right side lesion (of congenital heart disease) | | Q22.5 | Ebstein anomaly |
|  |  | Q22.4 | Congenital tricuspid stenosis |
|  |  | Q22.6 | Hypoplastic right heart syndrome |
|  |  | Q22.8 | Other congenital malformations of tricuspid valve |
|  |  | Q22.9 | Congenital malformation of tricuspid valve, unspecified |
|  |  | Q25.5 | Atresia of pulmonary artery |
|  |  | Q25.6 | Stenosis of pulmonary artery |
|  |  | Q25.7 | Other congenital malformations of pulmonary artery |
|  |  | Q22.0 | Pulmonary valve atresia |
|  |  | Q22.1 | Congenital pulmonary valve stenosis |
|  |  | Q22.2 | Congenital pulmonary valve insufficiency |
|  |  | Q22.3 | Other congenital malformations of pulmonary valve |
|  |  | Q24.3 | Pulmonary infundibular stenosis |
| Other lesion (of congenital heart disease) | | Q26.0 | Congenital stenosis of vena cava |
|  |  | Q26.8 | Other congenital malformations of great veins |
|  |  | Q26.9 | Congenital malformation of great vein, unspecified |
|  |  | Q23.8 | Other congenital malformations of aortic and mitral valves |
|  |  | Q23.9 | Congenital malformation of aortic and mitral valves, unspecified |
|  |  | Q24.2 | Cor triatriatum |
|  |  | Q24.5 | Malformation of coronary vessels |
|  |  | Q26.3 | Partial anomalous pulmonary venous connection |
|  |  | Q26.4 | Anomalous pulmonary venous connection, unspecified |
|  |  | Q26.0 | Congenital stenosis of vena cava |
|  |  | Q26.8 | Other congenital malformations of great veins |
|  |  | Q26.9 | Congenital malformation of great vein, unspecified |
|  |  | Q26.2 | Total anomalous pulmonary venous connection |
| Cardiac arrest | | I46 | Cardiac arrest |
| Cardiomyopathy | | I42 | Cardiomyopathy |
| Congestive heart failure | | I11.0 | Hypertensive heart disease with (congestive) heart failure |
|  |  | I13.0 | Hypertensive heart and renal disease with (congestive) heart failure |
|  |  | I13.2 | Hypertensive heart and renal disease with both (congestive) heart failure and renal failure |
|  |  | I50 | Heart failure |
| Endocarditis | | I33 | Acute and subacute endocarditis |
|  |  | I38 | Endocarditis, valve unspecified |
|  |  | I39 | Endocarditis and heart valve disorders in diseases classified elsewhere |
| Arrhythmia | | I44 | Atrioventricular and left bundle-branch block |
|  |  | I45 | Other conduction disorders |
|  |  | I47 | Paroxysmal tachycardia |
|  |  | I48 | Atrial fibrillation and flutter |
|  |  | I49 | Other cardiac arrhythmias |
| Conduction disorder  (Subgroup of arrhythmia) | | I44.0 | Atrioventricular block, first degree |
|  |  | I44.1 | Atrioventricular block, second degree |
|  |  | I44.2 | Atrioventricular block, complete |
|  |  | I44.3 | Other and unspecified atrioventricular block |
|  |  | I45.5 | Other specified heart block (Sinoatrial block, Sinoauricular block) |
|  |  | I45.8 | Other specified conduction disorders (Atrioventricular [AV] dissociation, Interference dissociation) |
|  |  | I45.9 | Conduction disorder, unspecified |
| WPW (Subgroup of arrhythmia) | | I45.6 | Pre-excitation syndrome |
| SVT (Subgroup of arrhythmia) | | I47.1 | Supraventricular tachycardia |
| AL/AFL (Subgroup of arrhythmia) | | I48 | Atrial fibrillation and flutter |
| VA (Subgroup of arrhythmia) | | I47.0 | Re-entry ventricular arrhythmia |
|  |  | I47.2 | Ventricular tachycardia |
|  |  | I49.0 | Ventricular fibrillation and flutter |
| SSS (Subgroup of arrhythmia) | | I49.5 | Sick sinus syndrome |
| Ischemic heart disease | | I20 | Angina pectoris |
|  |  | I21 | Acute myocardial infarction |
|  |  | I22 | Subsequent myocardial infarction |
|  |  | I23 | Certain current complications following acute myocardial infarction |
|  |  | I24 | Other acute ischemic heart diseases |
|  |  | I25 | Chronic ischemic heart disease |
| Pulmonary embolism | | I26 | Pulmonary embolism |
|  |  | O88.2 | Obstetric (pulmonary) embolism |
|  |  | O08.2 | Embolism following abortion and ectopic and molar pregnancy |
|  |  | O03.2 | Incomplete spontaneous abortion, complicated by embolism |
|  |  | O03.7 | Complete or unspecified spontaneous abortion, complicated by embolism |
|  |  | O06.2 | Incomplete unspecified abortion, complicated by embolism |
|  |  | O05.2 | Incomplete other abortion, complicated by embolism |
|  |  | O07.7 | Other and unspecified failed attempted abortion, complicated by embolism |
|  |  | O04.2 | Incomplete medical abortion, complicated by embolism |
|  |  | O04.7 | Complete or unspecified medical abortion, complicated by embolism |
|  |  | O05.7 | Complete or unspecified other abortion, complicated by embolism |
|  |  | O88 | Obstetric embolism |
| Sepsis | | A40 | Streptococcal sepsis |
|  |  | A41 | Other sepsis |
|  |  | A42 | Actinomycosis |
|  |  | A32 | Listeriosis |
|  |  | B37 | Candidiasis |
|  |  | O85 | Puerperal sepsis |
| Stroke | | I60 | Subarachnoid hemorrhage |
|  |  | I61 | Intracerebral hemorrhage |
|  |  | I62 | Other nontraumatic intracranial hemorrhage |
|  |  | I63 | Cerebral infarction |
| Hyperlipidemia | | E78 | Disorders of lipoprotein metabolism and other lipidemias |
| Hypertension | Pre-gestational hypertension | I10 | Essential(primary) hypertension |
|  |  | I11 | Hypertensive heart disease |
|  |  | I12 | Hypertensive renal disease |
|  |  | I13 | Hypertensive heart and renal disease |
|  |  | I15 | Secondary hypertension |
|  | Chronic hypertension | O10 | Pre-existing hypertension complicating pregnancy, childbirth and the puerperium |
|  | Superimposed preeclampsia | O11 | Pre-eclampsia superimposed on chronic hypertension |
| Diabetes | | E10 | Type 1 diabetes mellitus |
|  |  | E11 | Type 2 diabetes mellitus |
|  |  | E12 | Malnutrition-related diabetes mellitus |
|  |  | E13 | Other specified diabetes mellitus |
|  |  | E14 | Unspecified diabetes mellitus |
|  |  | O24.0 | Pre-existing type 1 diabetes mellitus |
|  |  | O24.1 | Pre-existing type 2 diabetes mellitus |
|  |  | O24.2 | Pre-existing malnutrition-related diabetes mellitus |
|  |  | O24.3 | Pre-existing diabetes mellitus, unspecified |
| Hypertension during pregnancy | Gestational hypertension | O13 | Gestational [pregnancy-induced] hypertension |
|  | Preeclampsia | O14 | Pre-eclampsia |
|  | Eclampsia | O15 | Eclampsia |
|  | Unspecified maternal hypertension | O16 | Unspecified maternal hypertension |
| Gestational diabetes | | O24.4 | Diabetes mellitus arising in pregnancy |
|  |  | O24.9 | Diabetes mellitus in pregnancy, unspecified |

PTB = preterm birth; PPROM = preterm premature rupture of membranes; PTL = preterm labor; WPW = Wolff-Parkinson-White syndrome; SVT = supraventricular tachycardia; AF = atrial fibrillation; AFL = atrial flutter; VA = ventricular arrhythmia; SSS = sick sinus syndrome.
