# Supplementary material for: Preterm birth and maternal heart disease: A machine learning analysis using the Korean national health insurance database
Source: PLoS One. 2023 Mar 31;18(3):e0283959. doi: 10.1371/journal.pone.0283959 (PMC10065252; doi:10.1371/journal.pone.0283959)
Supplement: S2 Table — (DOCX) [file pone.0283959.s002.docx]

**S2 Table. ATC code for medication**

| **Code** | **Medication** |
| --- | --- |
| N05BA | Benzodiazepine |
| N05CD |  |
| N05CF |  |
| C08 | Calcium channel blocker |
| C01DA | Nitrate |
| G03 | Progesterone |
| N05C | Hypnotics and sedatives |
| N06A | Tricyclic antidepressant |

ATC = Anatomical Therapeutic Chemical
