# Supplementary material for: Preterm birth and maternal heart disease: A machine learning analysis using the Korean national health insurance database
Source: PLoS One. 2023 Mar 31;18(3):e0283959. doi: 10.1371/journal.pone.0283959 (PMC10065252; doi:10.1371/journal.pone.0283959)
Supplement: S3 Table — (DOCX) [file pone.0283959.s003.docx]

|  | Total | Term birth | Preterm birth |
| --- | --- | --- | --- |
| Pregnant women with heart disease |  |  |  |
| Arrhythmia | 7,312 | 6,713 (91.8) | 599 (8.2) |
| Ischemic heart disease | 5,007 | 4,565 (91.2) | 442 (8.8) |
| Cardiomyopathy | 94 | 79 (84.0) | 15 (16.0) |
| Congestive heart failure | 846 | 756 (89.4) | 90 (10.6) |
| Cyanotic CHD | 42 | 36 (85.7) | 6 (14.3) |
| Acyanotic CHD | 292 | 266 (91.1) | 26 (8.9) |
| Pregnant women without heart disease | 161,333 | 149,810 (92.9) | 11,523 (7.1) |

**S3 Table. The prevalence of PTB in pregnant woman with and without heart disease**

CHD = congenital heart disease.
