# Supplementary material for: Preterm birth and maternal heart disease: A machine learning analysis using the Korean national health insurance database
Source: PLoS One. 2023 Mar 31;18(3):e0283959. doi: 10.1371/journal.pone.0283959 (PMC10065252; doi:10.1371/journal.pone.0283959)
Supplement: S4 Table — (DOCX) [file pone.0283959.s004.docx]

**S4 Table. Variable importance in random forest prediction model for PTB 1–3**

| **Rank** | **PTB1** | **Variable importance** | **PTB2** | **Variable importance** | **PTB3** | **Variable importance** |
| --- | --- | --- | --- | --- | --- | --- |
| 1 | Socioeconomic status | 0.3363 | Socioeconomic status | 0.3210 | Socioeconomic status | 0.3446 |
| 2 | Age | 0.2774 | Age | 0.2498 | Age | 0.2781 |
| 3 | Gestational diabetes | 0.0402 | Sepsis | 0.0397 | Gestational diabetes | 0.0395 |
| 4 | Anemia | 0.0361 | Gestational diabetes | 0.0389 | Anemia | 0.0357 |
| 5 | Abnormal menstruation | 0.0327 | Benzodiazepine | 0.0367 | Sepsis | 0.0328 |
| 6 | Sepsis | 0.0309 | Abnormal menstruation | 0.0346 | Abnormal menstruation | 0.0291 |
| 7 | Progesterone | 0.0253 | Anemia | 0.0339 | Benzodiazepine | 0.0261 |
| 8 | Benzodiazepine | 0.0234 | Pelvic inflammatory disease | 0.0315 | Hypertension | 0.0222 |
| 9 | Hypertension | 0.0225 | Hyperlipidemia | 0.0268 | Hyperlipidemia | 0.0213 |
| 10 | Pelvic inflammatory disease | 0.0218 | Hypertension | 0.0244 | Tricyclic antidepressant | 0.0212 |
| 11 | Hyperlipidemia | 0.0207 | Vaginitis | 0.0228 | Vaginitis | 0.0200 |
| 12 | Tricyclic antidepressant | 0.0206 | Progesterone | 0.0224 | Progesterone | 0.0195 |
| 13 | Vaginitis | 0.0188 | Tricyclic antidepressant | 0.0219 | Pelvic inflammatory disease | 0.0184 |
| 14 | Arrhythmia | 0.0144 | Arrhythmia | 0.0145 | Arrhythmia | 0.0147 |
| 15 | Hypertension during pregnancy | 0.0135 | Recurrent miscarriage or infertility | 0.0143 | Diabetes | 0.0115 |
| 16 | Diabetes | 0.0116 | Hypnotic/sedative drug | 0.0134 | Hypnotic/sedative drug | 0.0114 |
| 17 | Recurrent miscarriage or infertility | 0.0111 | Diabetes | 0.0118 | Recurrent miscarriage or infertility | 0.0108 |
| 18 | Hypnotic/sedative drug | 0.0105 | Endometriosis | 0.0110 | Ischemic heart disease | 0.0106 |
| 19 | Ischemic heart disease | 0.0105 | Ischemic heart disease | 0.0106 | Hypertension during pregnancy | 0.0094 |
| 20 | Endometriosis | 0.0086 | Hypertension during pregnancy | 0.0080 | Endometriosis | 0.0089 |
| 21 | Stroke | 0.0031 | Stroke | 0.0028 | Stroke | 0.0032 |
| 22 | Congestive heart failure | 0.0029 | Congestive heart failure | 0.0025 | Congestive heart failure | 0.0031 |
| 23 | Calcium channel blocker | 0.0019 | Calcium channel blocker | 0.0017 | Calcium channel blocker | 0.0020 |
| 24 | Nitrate | 0.0012 | Nitrate | 0.0011 | Nitrate | 0.0012 |
| 25 | Pelvic organ prolapse | 0.0011 | Pelvic organ prolapse | 0.0008 | Pelvic organ prolapse | 0.0012 |
| 26 | Cardiomyopathy | 0.0009 | Cardiomyopathy | 0.0007 | Cardiomyopathy | 0.0010 |
| 27 | Acyanotic CHD | 0.0007 | Acyanotic CHD | 0.0005 | Acyanotic CHD | 0.0007 |
| 28 | Shunt lesion | 0.0004 | Shunt lesion | 0.0005 | Pulmonary embolism | 0.0004 |
| 29 | Pulmonary embolism | 0.0003 | Pulmonary embolism | 0.0004 | Shunt lesion | 0.0004 |
| 30 | Cyanotic CHD | 0.0002 | Severe lesion | 0.0004 | Severe lesion | 0.0002 |
| 31 | Lt. side lesion | 0.0002 | Lt. side lesion | 0.0003 | Cyanotic CHD | 0.0002 |
| 32 | Severe lesion | 0.0001 | Cyanotic CHD | 0.0001 | Lt. side Lesion | 0.0001 |
| 33 | Endocarditis | 0.0001 | Endocarditis | 0.0000 | Endocarditis | 0.0001 |
| 34 | Rt. side lesion | 0.0001 | Rt. side lesion | 0.0000 | Rt. side lesion | 0.0001 |
| 35 | Other lesion | 0.0001 | Other lesion | 0.0000 | Other lesion | 0.0001 |
| 36 | Cardiac arrest | 0.0000 | Cardiac arrest | 0.0000 | Cardiac arrest | 0.0000 |

PTB 1 - PTB with preterm premature rupture of membranes (PPROM) only; PTB 2 - PTB with spontaneous preterm labor without PPROM; PTB 3 - PTB 1 or PTB 2

PTB = preterm birth; CHD = congenital heart disease; Lt. = left; Rt. = right.
