# Supplementary material for: Preterm birth and maternal heart disease: A machine learning analysis using the Korean national health insurance database
Source: PLoS One. 2023 Mar 31;18(3):e0283959. doi: 10.1371/journal.pone.0283959 (PMC10065252; doi:10.1371/journal.pone.0283959)
Supplement: S5 Table — (DOCX) [file pone.0283959.s005.docx]

**S5 Table. SHAP range for PTB 4**

|  | **Min** | **Max** |
| --- | --- | --- |
| Congestive heart failure | -0.0090 | 0.3211 |
| Hypertension during pregnancy | -0.0872 | 0.2724 |
| Diabetes | -0.0957 | 0.2501 |
| Cardiomyopathy | -0.0039 | 0.2408 |
| Age | -0.1234 | 0.2318 |
| Ischemic heart disease | -0.0357 | 0.2289 |
| Socioeconomic status | -0.1388 | 0.2202 |
| Recurrent miscarriage or infertility | -0.0800 | 0.2016 |
| Endometriosis | -0.0888 | 0.2006 |
| Pelvic organ prolapse | -0.0310 | 0.1968 |
| Nitrate | -0.0182 | 0.1853 |
| Hyperlipidemia | -0.1332 | 0.1852 |
| Hypnotic/sedative drug | -0.0991 | 0.1781 |
| Abnormal menstruation | -0.0961 | 0.1765 |
| Pelvic inflammatory disease | -0.1295 | 0.1664 |
| Calcium channel blocker | -0.0036 | 0.1609 |
| Hypertension | -0.1565 | 0.1575 |
| Tricyclic antidepressant | -0.1262 | 0.1574 |
| Acyanotic CHD | -0.0225 | 0.1486 |
| Anemia | -0.1554 | 0.1460 |
| Progesterone | -0.1464 | 0.1384 |
| Sepsis | -0.1459 | 0.1324 |
| Shunt lesion | -0.0028 | 0.1320 |
| Gestational diabetes | -0.1153 | 0.1304 |
| Arrhythmia | -0.1246 | 0.1263 |
| Vaginitis | -0.1384 | 0.1224 |
| Benzodiazepine | -0.1207 | 0.1192 |
| Stroke | -0.0655 | 0.1092 |
| Pulmonary embolism | -0.0359 | 0.0484 |
| Severe lesion | -0.0075 | 0.0314 |
| Cyanotic CHD | -0.0436 | 0.0215 |
| Right side lesion | -0.0446 | 0.0138 |
| Left side lesion | -0.0216 | 0.0028 |
| Endocarditis | -0.0618 | 0.0028 |
| Cardiac arrest | -0.0389 | 0.0025 |
| Other lesions | 0.0000 | 0.0000 |

PTB 4 indicated PTB with preterm premature rupture of membranes or spontaneous preterm labor or other indicated PTB due to maternal or fetal indications.

SHAP = shapley additive explanation; PTB = preterm birth; CHD = congenital heart disease.
