# Supplementary material for: Preterm birth and maternal heart disease: A machine learning analysis using the Korean national health insurance database
Source: PLoS One. 2023 Mar 31;18(3):e0283959. doi: 10.1371/journal.pone.0283959 (PMC10065252; doi:10.1371/journal.pone.0283959)
Supplement: S6 Table — (DOCX) [file pone.0283959.s006.docx]

**S6 Table. SHAP range of the arrhythmia subgroup**

|  | **Min** | **Max** |
| --- | --- | --- |
| AF/AFL | -0.0087 | 0.1576 |
| SVT | -0.1028 | 0.0163 |
| Conduction disorder | -0.0367 | 0.1496 |
| WPW | -0.0291 | 0.1286 |
| VA | -0.0373 | 0.0437 |
| SSS | 0.0000 | 0.0000 |

SHAP = shapley additive explanation; AF = atrial fibrillation; AFL = atrial flutter; SVT = supraventricular tachycardia; WPW = Wolff-Parkinson-White syndrome; VA = ventricular arrhythmia; SSS = sick sinus syndrome.
